# Successful Non-operative Management of Unstable Jefferson Fracture With Transverse Atlantal Ligament Injury (Dickman Type I and IIb) in a Pediatric Patient: A Case Report

**DOI:** 10.7759/cureus.67522

**Published:** 2024-08-22

**Authors:** Satoshi Hattori

**Affiliations:** 1 Department of Spinal Surgery, Hachioji Spine Clinic, Hachioji, JPN

**Keywords:** non-surgical management, non-operative treatment, somi brace, halo vest, upper cervical spine injury, children, atlantoaxial instability, avulsion fracture, transverse atlantal ligament, jefferson fracture

## Abstract

This report presents the case of a Jefferson fracture (posterior arch fracture) associated with an unstable avulsion fracture and substance injury of the transverse atlantal ligament (Dickman type I and IIb) in an eight-year-old male child. The patient was managed conservatively with external immobilization using a halo vest and a sternal occipital mandibular immobilizer (SOMI) brace and subsequently made a full recovery. Computed tomography (CT) and dynamic cervical spine radiographs at the final follow-up demonstrated solid reattachment of the avulsed bony fragment of the transverse atlantal ligament and no instability at the C1/2 level. This case report adds to the literature on the optimal non-operative management of the rare pediatric unstable C1-C2 trauma.

## Introduction

Cervical spine injuries are uncommon in children, accounting for only 1.5% of all injured children in the National Pediatric Trauma Registry, United States [1]. Cases involving the upper cervical spine are more prevalent in younger children, specifically those under the age of eight, compared to older children. Isolated C1 fractures, also known as Jefferson fractures, are exceedingly uncommon in children. Furthermore, an accurate diagnosis at the initial presentation in children is frequently impeded by communication challenges, a lack of evidence for these uncommon pathologies, and various pitfalls in imaging of the pediatric cervical spine, including pseudosubluxation, synchondroses, an increased normal range of atlantodental distance, and a normal "wedge appearance " of the vertebrae [2-7].

Jefferson fractures are usually stable in children due to the integrity of the transverse atlantal ligament (TAL), allowing successful management with external immobilization [2,7]. There are only a few reports of Jefferson fractures with concomitant unstable TAL injuries in young children with immature spines [8,9]. The advent of advanced magnetic resonance imaging (MRI) and computed tomography (CT) technology has greatly facilitated the assessment of the anatomical and functional integrity of the upper cervical spine in the pediatric population [3,10]. 

This report presents a case of a type I Jefferson fracture (posterior arch fracture according to the Landells classification) [11,12] associated with simultaneous avulsion and substance injury of the TAL (Dickman type I and IIb) in a young child. The patient was treated with a halo vest and sternal occipital mandibular immobilizer (SOMI) brace and subsequently fully recovered without instability of the C1/2 segment. This case study will provide important insight into the optimal non-operative management of these rare pathologies.

## Case presentation

History and examination

**Figure 1:**
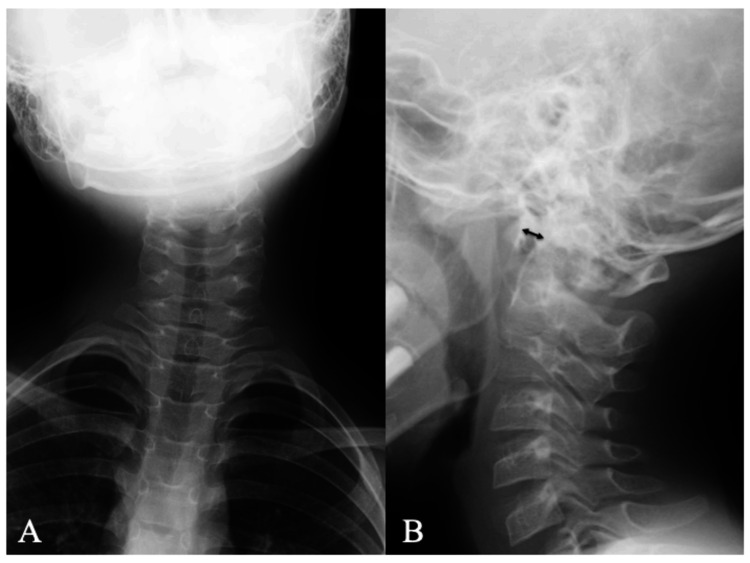
Cervical spine X-rays at initial presentation Plain radiograph of the cervical spine at initial presentation showing (A) the right torticollis and (B) enlargement of the atlantodental interval (6 mm, double-headed arrow) in the neutral lateral cervical spine position.

An eight-year-old boy was admitted to the hospital via outpatient clinic a day after hitting his forehead on the bottom of the swimming pool while diving into the water. He complained of severe upper cervical pain, tenderness, torticollis, and limited cervical range of motion (ROM). Fortunately, he had no neurological deficits in either the spinal cord or cranial nerves. Radiographs of the cervical spine showed right-sided torticollis and enlargement of the atlantodental interval (ADI) (6 mm on neutral lateral radiograph; Figure 1) [13]. 

**Figure 2:**
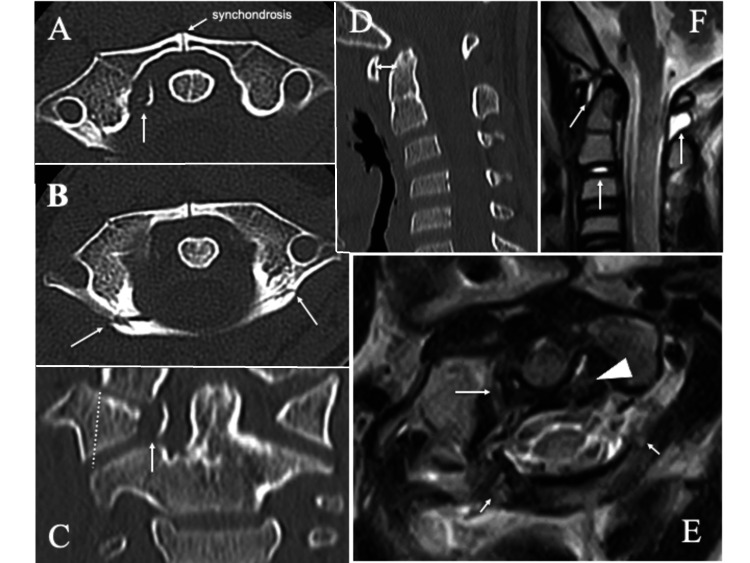
CT and MRI findings at initial presentation CT at initial presentation showing (A, C) an avulsion fracture of the right transverse atlantal ligament (arrows), (B) bilateral posterior arch fractures of the atlas (arrows), (C) slight lateral displacement of the right C1 lateral mass (dotted line), and (D) slight enlargement of the ADI (double-headed arrow). T2-weighted MRI showing (E) high signal intensity in the avulsed osseous tubercle of the right C1 lateral mass (arrow) and the fractured posterior atlantal arch (short arrows) and mild hyperintensity in the left substance of the transverse atlantal ligament (arrowhead) and (F) edematous or hemorrhagic changes around the odontoid process and in the cervical disc at C2/3 and in the posterior ligamentous complex at C1/2 (arrows). ADI: atlantodental interval

CT of the neck showed avulsion of the TAL from the right C1 lateral mass with a small displaced bone fragment (gap: 4.5 mm, Dickman type IIb TAL injury, avulsion type; Figure 2a). There were also minimally displaced bilateral fractures of the posterior arch of the atlas (posterior arch fracture, Landells type I Jefferson fracture; Figure 2b) [12], a slight lateral displacement of the right C1 lateral mass (the lateral mass offset of Spence: 4 mm; Figure 2c), and slight enlargement of the ADI (Figure 2d) [14]. MRI (T2-weighted image) showed high signal intensity in both the avulsed osseous tubercle of the right C1 lateral mass and the fractured posterior atlantal arch (Figure 2e). The MRI signal of the left substance of the TAL was mildly hyperintense, suggesting mild damage to the substance of the TAL on the opposite side of the avulsion, but the continuity of the ligament was preserved (Figure 2e). In addition, high signal changes on MRI were observed around the odontoid process, in the cervical disc at C2/3, and in the posterior ligamentous complex at C1/2, suggesting tissue edema or hemorrhage due to the injury (Figure 2f).

Treatment and follow-up

**Figure 3:**
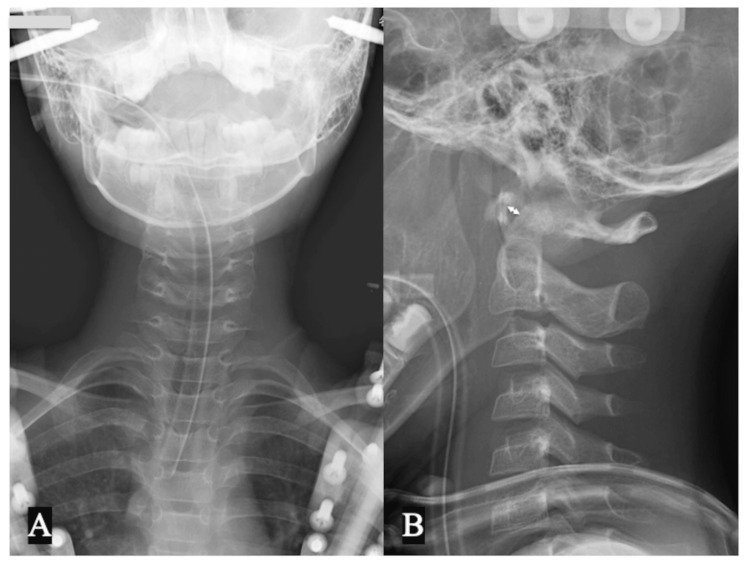
Cervical spine X-rays after halo vest application Plain radiograph of the cervical spine immediately after halo vest application showing a reduction in (A) the torticollis and (B) atlantoaxial subluxation; atlantodental interval 3 mm (double-headed arrow)

**Figure 4:**
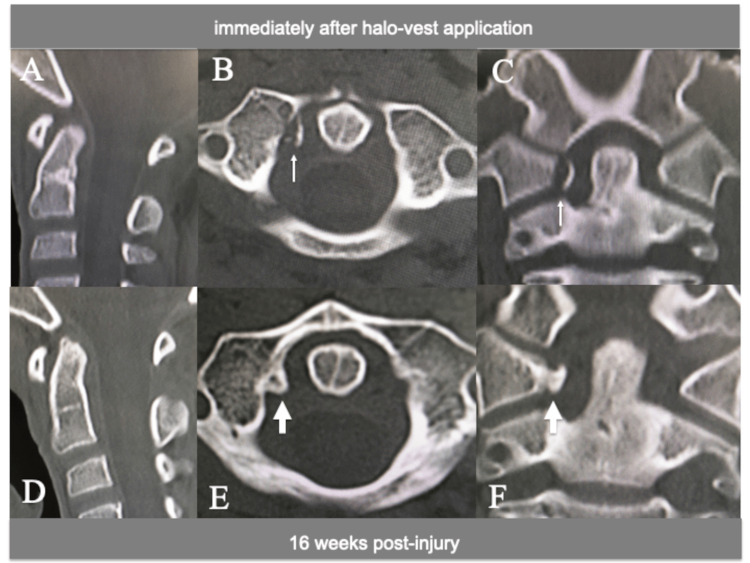
CT images immediately after halo-vest application and 16 weeks post injury CT showing (A, B, C) no increase in the displacement of the avulsed bone fragment immediately after halo vest application (arrows) and (D, E, F) solid fusion with the C1 lateral mass at 16 weeks post injury (large arrows).

**Figure 5:**
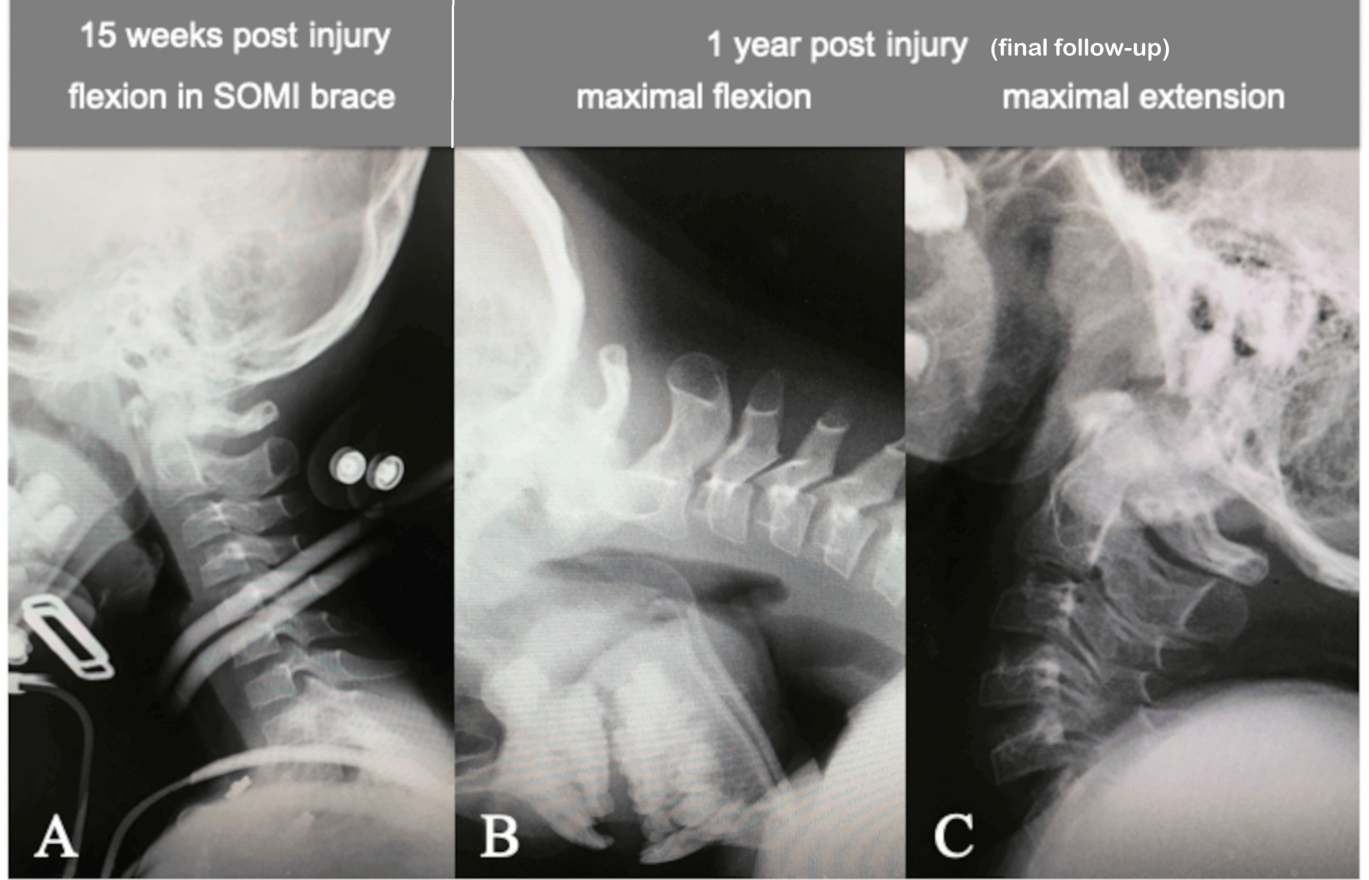
Cervical spine X-rays at 15 weeks post injury and at the one-year final follow-up Dynamic flexion-extension cervical spine radiographs showing (A) a normal atlantodental interval (3 mm) and no evidence of instability at 15 weeks post injury and (B, C) that the cervical ROM remained normal with no abnormal motion or instability at the final one-year follow-up. ROM: range of movement; SOMI: sternal occipital mandibular immobilizer

A pediatric halo vest was applied in the reduction position of the subluxated atlantoaxial joint (ADI 3 mm, Figure 3) under general anesthesia with endotracheal intubation, and the reduction status was monitored with periodic radiographs for eight weeks. The patient then underwent additional immobilization with a SOMI brace for eight weeks until CT scan demonstrated the solid fusion of the avulsed bone fragment of the TAL with the C1 lateral mass (Figure 4). Dynamic flexion-extension cervical spine radiographs showed a normal ADI and no evidence of instability at 15 weeks post injury (Figure 5a). The patient did not complain of any further neck pain, discomfort, or neurological deficits, and his cervical ROM remained normal, with no abnormal motion at the final one-year follow-up examination (Figure 5b-5c). The patient began ROM training in the rehabilitation unit at five months post injury and returned to his previous swimming sports activities at six months, including diving from the starting platform of the pool at 12 months.

## Discussion

Cervical spine injuries, including C1-C2 levels, are relatively less common in children than in adults, accounting for only 1-10% of all spine injuries [15]. Younger children are more likely to sustain upper cervical spine injuries because of the large proportion of the head in relation to the rest of the body, the flexibility of the immature spine, and the greater frequency of fulcrum cervical motion at C2-C3 [3,11]. While blunt trauma from motor vehicle accidents and falls are the most common causes of pediatric upper cervical spine injuries under the age of eight years, sports-related injuries are a greater cause of injury besides motor vehicle accidents above the age of eight years [1,4,15]. In the present case, the high-energy trauma caused by the axial load, in conjunction with the extension-lateral flexion/rotation force during the contusion of the frontal head on the swimming pool floor after diving, resulting in the distinctive combination of upper cervical spine injuries, including posterior arch fractures (Jefferson type I) and a unilateral avulsion fracture of the TAL (Dickman type IIb) [12,16]. Furthermore, MRI indicated incomplete substance damage to the TAL (Dickman type I) on the side opposite the avulsed TAL fracture. Fortunately, the continuity of the TAL substance was preserved, which may have contributed to the successful non-operative treatment with external immobilization. To the best of my knowledge, there are few reports of a Jefferson fracture associated with a concomitant unstable avulsion fracture and substance damage of the TAL in a young child.

The TAL plays a primary role in restraining the atlantoaxial joint, and its structural integrity is a crucial factor in predicting the prognosis of C1-C2 spinal injuries. The first report of avulsion fractures of the TAL in adult patients was published by Barker and colleagues [17]. Dickman et al. proposed a classification of TAL injuries into three types: type I, a ligamentous substance injury; type IIa, a burst fracture of the C1 lateral mass; and type IIb, an avulsion fracture of the C1 lateral mass [18]. Furthermore, they posited that type I injuries are unable to heal satisfactorily without internal fixation and should be treated with early surgery, and type II injuries should be treated initially with a rigid cervical brace for three to four months, as they have a 74% success rate non-operatively [18].

It is important to note that the guidelines for the management of TAL injuries according to subtype classification cannot be generalized to young children due to the limited number of reports relating to these pathologies in children. Lo et al. [8] and Vilela and Peterson [9] reported three pediatric cases of Dickman type IIb avulsion TAL injury and one pediatric case of complete TAL rupture with a Jefferson fracture, respectively. The patients were treated with external immobilization using halo vests or SOMI braces. The immobilization periods in the four cases were as follows: eight weeks in a SOMI brace, three months in a Halo vest, two months in a Halo vest followed by one month in a SOMI brace, and 11 weeks in a Halo vest, respectively. At the final follow-up, no evidence of instability was detected.

Although the lateral mass offset, as defined by Spence, was 4 mm and did not exceed 6.9 mm, which did not reach the instability criteria of the lateral atlantoaxial joint [14], the ADI (6 mm) was more than 5 mm in the neutral cervical position (Figures 1b, 2d). These radiological findings indicated the presence of atlantoaxial instability due to double TAL injuries, specifically TAL type I incomplete disruption on the left side and a type IIb displaced avulsion fracture on the right side. Accordingly, the author utilized a halo vest in a position that reduced the subluxated atlantoaxial joint (ADI: 3 mm) and maintained it for eight weeks, during which time no complications related to the halo vest were observed. Subsequently, a SOMI brace was applied for an additional eight weeks. At the four-month radiological follow-up, the avulsed bone fragment had undergone complete fusion with the lateral mass, thereby restoring the integrity of the TAL. The radiographs of the lateral cervical spine in flexion-extension demonstrated the absence of atlantoaxial instability.

The utilization of the halo vest in pediatric patients may result in a range of complications, including pin-site infection, pin loosening, pressure ulcers, persistent pain, and in rare instances, intracranial abscess [19]. Accordingly, in the current case, both a halo vest and a SOMI brace were employed for external immobilization to reduce the duration of halo vest use [8]. Further studies are necessary to determine the optimal treatment option for this rare condition in pediatric patients.

## Conclusions

This report presents a clinical outcome that suggests the efficacy of external immobilization using a halo vest and SOMI brace in the treatment of Jefferson fractures associated with a simultaneous unstable avulsion fracture and incomplete TAL substance damage (Dickman type I and IIb) in young children. It must be acknowledged that the present results regarding the efficacy of external immobilization cannot necessarily be applied as a standard treatment for more unstable pediatric Jefferson fractures associated with complete TAL damage. However, external fixation for a period of two to three months may become a reasonable primary treatment to prevent spinal fusion in the immature pediatric spine. Further research is required to determine the optimal management of these rare, unstable upper cervical injuries in children.
